# Antidiabetic Potential of Mangiferin: An In Silico and In Vivo Approach

**DOI:** 10.3390/pharmaceutics17101262

**Published:** 2025-09-26

**Authors:** Anna Vesnina, Violeta Le, Svetlana Ivanova, Alexander Prosekov

**Affiliations:** 1 Laboratory of Natural Nutraceuticals Biotesting, Kemerovo State University, Kemerovo 650043, Russia; koledockop1@mail.ru (A.V.); ya808@yandex.ru (V.L.); 2 Institute of NBICS-Technologies, Kemerovo State University, Krasnaya Street, 6, Kemerovo 650043, Russia; 3 Department of TNSMD Theory and Methods, Kemerovo State University, Krasnaya Street, 6, Kemerovo 650043, Russia; 4 Laboratory of Biocatalysis, Kemerovo State University, Kemerovo 650043, Russia; a.prosekov@inbox.ru

**Keywords:** mangiferin, in silico, *Hedysarum neglectum*, diabetes mellitus, anti-inflammatory and antidiabetic activity

## Abstract

**Objectives:** According to published data, mangiferin has the potential to prevent diabetes mellitus. The aim of this work was to obtain in vivo evidence of the biological activity of mangiferin predicted in silico. **Methods:** A prediction using the IT Microcosm system was employed to identify the correlation between the spatial structure of mangiferin and its biological activity. *MAPK10*, *HCAR2*, and *CALCRL* biotargets were used as the basis for predicting moderate antiglycation activity in silico. The presence of anti-inflammatory and antidiabetic activities in mangiferin was empirically tested in in vivo models. To assess anti-inflammatory activity in female Sprague–Dawley rats, acute exudative inflammation and chronic proliferative inflammation were induced. To assess hypoglycemic activity in female Sprague–Dawley rats, diabetes mellitus was modeled with an alloxan solution (150.0 mg/kg). During the experiment, fasting body weight, glucose, and total cholesterol concentrations in the blood serum of the animals were assessed weekly. To study hypocholesterolemic activity in female *Mus musculus* mice, hypercholesterolemia was modeled by administering a solution of Kolliphor P 407 three times a week. Mangiferin (50.0 mg/kg, 100.0 mg/kg) was administered orally daily for 7 days (in the last week of the experiment) or for 14 days (hypercholesterolemia model). **Results:** In vivo studies showed that mangiferin showed pro-inflammatory activity without affecting body weight and did not reduce glucose and cholesterol concentrations. The obtained results contribute to the evidence regarding the presence/absence of the anti-inflammatory, hypoglycemic, and hypocholesterolemic properties of mangiferin. **Conclusions:** The discrepancy between mangiferin’s actual activity and the in silico predictions suggests the need for further studies using lower doses of mangiferin and investigating approaches to enhance its bioavailability.

## 1. Introduction

A decline in healthy life expectancy is associated with the increasing prevalence of metabolic diseases (obesity, atherosclerosis and cardiovascular diseases, diabetes, non-alcoholic fatty liver disease, etc.) [1]. According to statistics, these conditions are among the leading causes of mortality worldwide [2,3].

**Figure 1:**
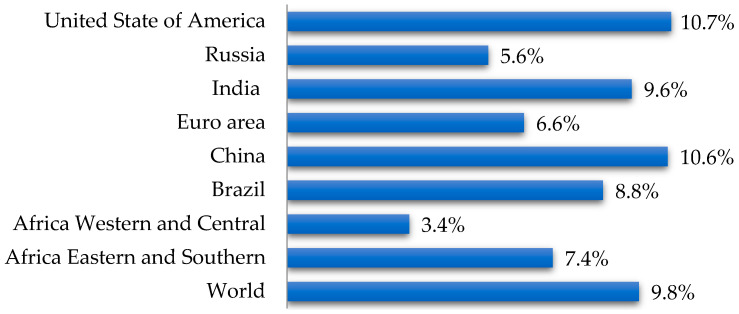
Distribution of type 1 diabetes worldwide (according to data [5]).

Diabetes mellitus (DM) is a common chronic disease associated with impaired metabolism and characterized by hyperglycemia, insulin deficiency, or impaired insulin utilization [4]. According to the World Bank [5], in 2021, 9.8% of the global population aged 20 to 79 years had DM (Figure 1).

Gregory and colleagues [6] reported that the global incidence of diabetes rose from 285 million to 537 million people between 2010 and 2021. Magliano et al. [7] further predicted that the global prevalence of diabetes will reach 643 million and 783 million people by 2030 and 2045, respectively.

**Figure 2:**
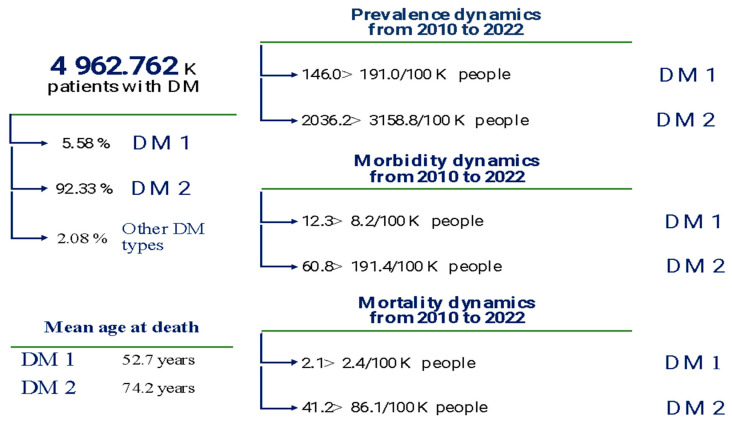
Epidemiological indicators of diabetes in the Russian population (2022): DM1—type 1 diabetes mellitus; DM2—type 2 diabetes mellitus (according to data [8,9,10]).

Although the Russian Federation is not among the leading countries in these statistics, the number of patients with type 2 diabetes remains significant. According to the Federal Register of Diabetes Mellitus [8] and related studies [9,10], as of January 1, 2023, the total number of diabetes patients under regular medical supervision accounted for 3.31% of Russia’s total population (Figure 2).

Fat accumulation (obesity), impaired glucose and cholesterol metabolism leading to insulin resistance, inflammatory diseases, and other diabetes mellitus risk factors serve as important markers for preventive measures [11,12].

When creating medications for the treatment and prevention of type 2 diabetes, synthetic or natural components are used, among which medicinal plant metabolites predominate [13,14,15]. Metabolites with established activity against diabetes symptoms include curcumin [16], luteolin [16], resveratrol [16], metformin [17], and others.

Similar substances include mangiferin (alpizarin or quinomine)—2-C-β-D-glucopyranosyl-1,3,6,7-tetraoxyxanthone—a xanthone glucoside. Its chemical formula is C19H18O11, and its average molecular weight is 422.34. There is evidence that it has antioxidant, immunostimulating, and cardioprotective activity, as well as the ability to control metabolic disorders [11,12,18,19]. The bioactivity of mangiferin is due to the presence of several functional groups: aromatic hydroxyl groups, non-aromatic hydroxyl groups, glycoside hydroxyl groups, and lactone carbonyl groups [20,21,22,23].

In vitro studies have demonstrated that mangiferin can inhibit alpha-glucosidase activity [24,25,26]. Zhang et al. [27] showed that mangiferin improves insulin resistance by regulating the level of free fatty acids in HepG2 and C2C12 cells. Additional research suggests potential antidepressant effects of mangiferin [28,29]. A study by Arora et al. [30] showed that mangiferin exhibits neuroprotective potential: it reduces memory deficit, gait disorders, motor misalignment, oxidative stress, and neuroinflammation and normalizes mitochondrial function in Wistar rats injected with quinoline acid, a neurotoxicant. Previous work by Frolova et al. [31] also reported mangiferin’s neuroprotective effects in *Caenorhabditis elegans* models. An in vivo experiment on rodents provided evidence that mangiferin in doses of 50 and 100 mg/kg does not exhibit mutagenic properties [32]. As part of the analysis of the published materials, it was found that mangiferin is a safe substance and has the potential to be used in the prevention of diabetes mellitus and other metabolic disorders.

The traditional source for mangiferin extraction is *Mango indica* Linnaeus leaves [33,34], which have antidiabetic, antioxidant, antimicrobial, antitumor, and anti-inflammatory properties [35]. However, there are limitations on the extraction of mangiferin from these plant raw materials, which include the content of the substance in the leaves, ranging from 5 to 12%, and the presence of a difficult-to-separate admixture of homoisomangiferin [36]. Research suggests that potential alternative sources, including *Anemarrhena asphodeloides* Bge., *Belamcanda chinensis* (L.) DC. (native to China) [37,38,39], and various *Hedysarum* species (Fabaceae family) [36,40,41,42], whose metabolites are often used to treat febrile diseases, fever, cough, and diabetes [43], have antibacterial, anti-inflammatory, antioxidant, and antitumor activity [44,45]. Due to their chemical composition, various *Hedysarum* species are actively used in Russia in folk medicine as a tonic and anti-inflammatory agent with choleretic activity [3,46].

Due to the modern development of science and technology, it is possible to predict the bioactivity of drugs, nutrients, and biologically active substances in in silico models, which is a fast, cheap research method that does not require the approval of an ethics committee [31,32,33,34,36,37,40,41,46,47,48,49], followed by empirical confirmation.

This work aimed to obtain in vivo confirmation of the biological activity of mangiferin predicted in in silico models. As a result of active pharmacological and clinical research, pharmaceutical preparations based on partially purified extracts of medicinal plants are often among the best-selling medicines globally. Taking into account the labor intensity and time required to establish full-fledged, industrially significant plantations, along with high demand, developments based on biotechnological approaches are an alternative option for obtaining valuable components produced by plant cell cultures. In the present study, the use of mangiferin isolated from *Hedysarum neglectum*, with a degree of purification of at least 95%, avoids the side effects caused by impurities in medicinal plant extracts. In comparison with existing studies [11,12,18,19] on the potential of mangiferin for the prevention of diabetes mellitus and on the assessment of toxicity and/or acceptable safe doses of these molecules, the present study of anti-inflammatory and antidiabetic properties focuses on mangiferin isolated from *Hedysarum neglectum* with a degree of purification of at least 95%, which not only contributes to the evidence base of global studies on the presence/absence of the anti-inflammatory, hypoglycemic, and hypocholesterolemic properties of mangiferin but also ensures the absence of the side effects usually caused by impurities in medicinal plant extracts.

## 2. Materials and Methods

The object of this study was mangiferin isolated from the extract of *Hedysarum neglectum* in previous studies by the authors [50]. It is a finely dispersed greenish-beige powder with a degree of purification of at least 95%.

### 2.1. In Silico Analysis

#### 2.1.1. Prediction of Mangiferin’s Targeted Activity Spectrum in the Microcosm BioS System and Identification of Relevant Biotargets

The prediction of the spectrum of the targeted activity of mangiferin was performed using Microcosm BioS 20.6.6 software [51] from the unified Microcosm IT software package [52,53], employing a structural similarity method with known compounds as standards.

Diabetes-related biotargets were identified through the Open Targets system [54] and QSAR-DB data in Microcosm BioS 20.6.6 [51].

The Tmax indicator reflected the degree of this type of activity in an untested compound, and the Ind (Index) indicator characterized the possible level of this activity [55], from −5 (“inactive”) to +5 (“very highly active”). According to the forecast results, compounds with an activity level of Ind > 1 were considered conditionally promising for this type of targeted activity.

#### 2.1.2. Selection of 3D Models of Relevant Biotargets and Identification of Their Binding Sites

When selecting valid 3D models of relevant biotargets, all experimental X-ray 3D models of *Homo sapiens*, reviewed in the UniProt knowledge base [56] and available in the PDBe database [57], were analyzed. The criteria for the validity of experimental 3D models of target proteins were the following: (1) length of the simulated amino acid sequence; (2) resolution; (3) number of fragments [53].

For each relevant target protein, the most valid 3D model containing at least one reference inhibitor or antagonist was selected to identify the binding-site positions. All 3D biotarget visualizations were created using Chem3D software (CambridgeSoft, v9.0, Cambridge, MA, USA) [58].

Three approaches were used to identify the specific binding sites in eight targets: based on information about point amino acid mutations in proteins; based on amino acids that cause ligand binding in experimental 3D models of proteins detected by LigPlot+ software (EMBL-EBI, v1.4.5, Hinxton, UK) [59]; and based on information from molecular modeling of ligand binding to the corresponding proteins. This made it possible to reliably determine the localization of sites and their key binding amino acids.

#### 2.1.3. Construction of Optimized 3D Models of the Studied Compound

For the studied compound, 10 conformers with the lowest energy were constructed with MarvinSketch software (ChemAxon Ltd., 17.1.23, Budapest, Hungary) using molecular mechanics methods [60]. The constructed conformers were optimized with MOPAC2012 software (Molecular Sciences Software Institute, 12.301 W, Blacksburg, VA, USA) [61] using the semi-empirical quantum-chemical PM7 method.

#### 2.1.4. Ensemble Docking of the Studied Compound into the Binding Sites of Relevant Biotargets and Determination of the Most Affine to the Studied Compound

The ensemble docking of the optimized 3D models of the studied compound into specific sites of valid 3D models of the relevant target proteins was carried out using PyRx 0.8 (pyrx.sourceforge.io) [62,63] and AutoDock Vina (Scripps Research Institute, 1.1.2, La Jolla, CA, USA) software [64], taking into account the data on the key amino acids of the binding sites and the 3D models of the docking spaces covering the sites.

The target proteins most affine to the studied compound were determined based on the values of the minimum docking energy, ΔE, of the studied natural compound at specific binding sites of the relevant biotargets. Targets for which the value of the minimum docking energy was ΔE ≤ −9.0 kcal/mol, or targets for which the values of the minimum docking energy, ΔE, were quite different from all other indicators, were considered affine to a particular compound.

#### 2.1.5. Analysis of the Molecular Mechanism of Binding of the Studied Compounds

The analysis of the molecular mechanism of the binding of the studied compound to specific sites of the most affine biotargets was performed using LigandScout software (GmbH, 4.2.1, Vienna, Austria) [65,66].

#### 2.1.6. Consensus Prediction of Antiglycation Activity in the IT Microcosm System

The pharmacological activity levels of organic compounds were predicted from their structural formulas using the IT Microcosm 7.3 system, which implemented three decision-making strategies (conservative, normal, and risk) [51,52].

The conservative strategy takes into account the most stable patterns characteristic of this particular type of activity. The normal strategy provides for a choice of four forecasting methods, selecting the most accurate one. The risk strategy allows for indirectly considering the features associated with the possible mechanism of the effects and takes into account the novelty of the predicted structure to the maximum degree of the three strategies.

The antiglycation activity levels of the studied compounds were calculated with the Testing 7.3 software in the IT Microcosm 7.3 system using three prediction strategies [51,52]. For each of them, the spectra of the predictive estimates were translated into consensus estimates of the activity level: High, Moderate, Low, Inactive.

### 2.2. In Vivo Experiments

#### 2.2.1. Design

The anti-inflammatory properties of mangiferin were studied in SPF (Specific Pathogen-Free) female rats (*Rattus* sp.), while hypoglycemic and hypocholesterolemic activity were studied in SPF male rats and SPF CD-1 stock mice (*Mus musculus*), respectively, since males do not have an estrous cycle, the stage of which determines susceptibility to etiological factors in females [67]. The design of the experiment is presented in Table 1, and it was approved by the Bioethical Commission of Ifar LLC (Protocol/Application No. 181-182/2023, 29 September 2023, Tomsk, Russia). In all series of experiments, mangiferin was injected into the stomach of model animals at effective doses of 50.0 mg/kg and 100.0 mg/kg, while purified, freshly prepared water was used as a negative control [22].

Housing. The animals were kept in a barrier-type section for SPF animals. The following microclimate parameters were maintained in the animal husbandry room: air temperature, 20–26 °C; relative humidity, 30–70%; air exchange, 15 volumes/hour; and adjustable light regime, (12:12) h. The microclimate parameters were monitored, and the corresponding values were recorded twice a day.

**Figure 3:**
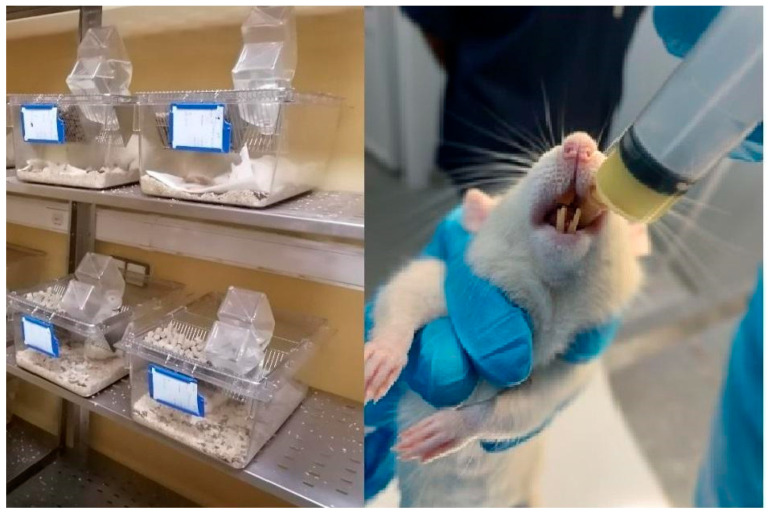
Vivarium with laboratory animals used for in vivo studies of mangiferin characteristics (photo from the KemSU collection).

Cells. The animals were kept in groups in T4 (rats)- and/or T2 (mice)-type cages. The cages were equipped with stainless steel lattice lids with a recess for feed and drinkers (Figure 3). The contaminated cells and cell equipment were replaced, disinfected, and sterilized once a week.

Bedding. Pre-sterilized, dry (no more than 15% humidity), dust-free wood shavings (Unikorm LLC, Tomsk Region, Russia), batch No. 03 dated May 2025, were used as bedding material.

Feed. In Models A (acute inflammation “carrageenan edema”) and B (acute inflammation “cotton granuloma”), the animals, except for those deprived of food according to the study plan, had unlimited access to full-grain granulated feed “Delta Feeds” for laboratory rats and mice (GOST 34566-2019, JSC “BioPro”, Novosibirsk Region, Dvurechye settlement), series 750/23 dated 18 May 2023. In Models C (diabetes mellitus) and D (hypercholesterolemia), the animals had unlimited access to full-grain granulated Delta Feeds for laboratory rats and mice (GOST 34566-2019, JSC BioPro, Novosibirsk region, Dvurechye settlement). The feed batches used were 1066/23 and 1306/23 (manufactured in 2023). The pre-sterilized feed was placed in the feed recess of the lid.

Water. The animals had constant access to drinkers with drinking water (polypropylene bottles with silicone stoppers with a stainless-steel spout). The water was prepared using the RO 588W-220-EZ reverse osmosis system (Raifil, Moscow, Russia).

Distribution by groups. The animals were randomly assigned to groups so that the spread of the initial weight did not exceed ±10% of the average value.

Identification. Each group was kept in a separate cage with an identification card label, indicating the following information: study number, stock name, decade of birth, gender, and number of animals. Individual identification was carried out using the tail-tag system.

Deprivation of food. On the eve of intragastric administration of the test substances in Model A (acute inflammation “carrageenan edema”), the animals were deprived of food for 18 h but had free access to drinking water. In Models C (diabetes mellitus) and D (hypercholesterolemia), the animals were deprived of food for 16 h before each blood sampling.

Animal welfare and mortality during the study. During the study of Models A and B, no animals were in critical condition, and none died. During the study of Models C and D, 16 rats died from alloxan toxicity, which was related to the features of the model used [68]. For all deceased animals, the time of death was recorded with maximum precision, and necropsies were performed within one day post-mortem.

Appearance, euthanasia. Rats were euthanized by CO_2_ inhalation with subsequent cervical dislocation, and mice were anesthetized with CO_2_ for blood collection and then euthanized. To assess general health and detect signs of toxicity, the identification of critical conditions and mortality of animals was carried out twice a day (in the morning and in the afternoon) and once a day on weekends and holidays.

Biochemistry. In Models C and D, blood was taken from the tail vein of rats, from which blood serum was obtained, and the concentrations of glucose and total cholesterol were determined. Blood was taken from the inferior vena cava of mice, from which blood serum was obtained, and the concentration of total cholesterol was determined. The measurements were carried out on a biochemical analyzer (Minitecno LIND 126 I.S.E. S.r.l., Rome, Italy) using commercial kits (Vector-Best, Novosibirsk, Russia).

#### 2.2.2. Anti-Inflammatory Activity In Vivo

When studying the anti-inflammatory properties of mangiferin, Model A of acute exudative inflammation (“carrageenan edema”) and Model B of chronic proliferative inflammation (“cotton granuloma”) [66] were used, which were implemented on 40 female SPF (Specific Pathogen-Free) rats (*Rattus* sp.) of SD (Sprague–Dawley) stock. The age of the rats at the beginning of the study was 20–24 weeks, and the body weight range was 300–411 g. In the study of the anti-inflammatory properties of mangiferin, female mice and rats were used, since they have a higher standardized body weight and more stable behavior than males [69].

In the experiment with carrageenan edema, 20 Sprague–Dawley rats were used; each group included 5 animals (Table 1, Groups 1–4). The animals in Group 1 served as a negative control and received water as part of the experiment. Diclofenac sodium (Chemopharm LLC, Obninsk, Russia), a nonsteroidal anti-inflammatory drug at an effective dose of 10.0 mg/kg, was used as the reference drug (positive control). Animals deprived of food for a period of no more than 18 h were injected with the test substance or diclofenac sodium preparation in a volume of no more than 1.0 ml. Paw edema was induced after 1 hour by subcutaneous injection of 0.1 ml of an aqueous 1% solution of λ-carrageenin (Sigma-Aldrich, St. Louis, MO, USA). Paw edema was assessed plethysmometrically 3 and 4 h after the induction of inflammation. A digital dry plethysmometer (OpenScience, Moscow, Russia) was used for measurement. The volume of liquid displaced by immersing the animal’s paw in a tube with liquid was recorded by a special sensor and displayed on a graphical LCD with an accuracy of 0.01 ml = 10 μL. The difference between the volume of the “control” and “experimental” paws, expressed in ml, determined the magnitude of the reaction [67].

In the experiment with chronic proliferative inflammation “cotton granuloma”, 20 Sprague–Dawley rats were used, with each group containing 5 animals (Table 1, Groups 5–8). The animals in Group 5 were a negative control and received water as part of the experiment. Diclofenac sodium was used as the reference drug (positive control). Within 7 days from the implantation of cotton swabs, the test substances were injected; on the 8th day, the wet granulomas were removed, weighed, and dried. On day 9, the granulomas were re-weighed. The proliferative reaction was assessed by the difference between the mass of the dried granuloma and the initial mass of the cotton swab. The exudative reaction was assessed by the difference between the mass of the wet and dried granuloma [67].

#### 2.2.3. Antidiabetic Activity In Vivo

When studying the antidiabetic properties of mangiferin, Model C of diabetes mellitus and Model D of hypercholesterolemia were used as part of diabetes mellitus [66], which were implemented in SPF male rats (*Rattus* sp.) and SPF male mice (*Mus musculus*) since they do not have an estrous cycle, the stage of which determines susceptibility to etiological factors in females. The body weight range at the beginning of the study was 219–272 g for rats and 40.8–49.6 g for mice. The age at the beginning of the study was 12 weeks for rats and 19 weeks for mice.

In the experiment to assess hypoglycemic activity, 50 rats were used, divided into 9 groups of 10 rats (Table 1, Groups 9–13). Group 9 was a group of intact animals (healthy animals that were kept and fed under general conditions, without treatment). The animals in Group 10 were a negative control and received water as part of the experiment. The positive control drug was glibenclamide (GLB), a commercial sugar-lowering drug, at a concentration of 5 mg (OZON Pharm, Tolyatti, Russia). Alloxan (Diaem LLC, Moscow, Russia) and poloxamer (Kolliphor) (BASF Corporation, Florham Park, NJ, USA) were used as pathology inducers. In animals from Groups 10–13, previously deprived of food for 16 h, diabetes mellitus was induced with a single intraperitoneal injection of alloxan solution at a dose of 150.0 mg/kg in a volume of 1.0 mL [68]. The body weight of the animals and the concentration of glucose in the blood were measured before and 48 h after the administration of alloxan to confirm the development of diabetes mellitus. Further, groups were formed only from animals with a blood glucose concentration of at least 11 mmol/L. To stabilize the condition of the animals, the test substance was administered after 2 weeks. The test substance was administered to rats once a day for 7 days in the last week of the experiment. The fasting body weight of the animals, the glucose concentration, and the total cholesterol in the blood were assessed once a week for 21 days. The hypoglycemic agent glibenclamide at an effective dose of 5.0 mg/kg [70] was administered intragastrically using the same scheme as mangiferin.

#### 2.2.4. Hypocholesterolemic Activity In Vivo

In the experiment to assess hypocholesterolemic activity, 29 CD-1 stock mice were used [71,72,73]. The intact group (Group 14) included 5 animals (healthy animals kept under general conditions), and the experimental groups each included 8 animals (Table 1, Groups 14–17). The animals in Group 15 served as a negative control. To simulate hypercholesterolemia for 2 weeks, the animals in Groups 15–17 were injected intraperitoneally with an aqueous solution of a lipoprotein lipase inhibitor that disrupts lipoprotein clearance, poloxamer P 407, at a dose of 400.0 mg/kg in a volume of 1.0 mL three times a week (Monday, Wednesday, and Friday). The intact animals in Group 14 did not receive any substances throughout the study. The studied substances and the negative control substance (water) were injected into the stomach of animals daily for 2 weeks in a volume of 1.0 mL [67]. After the last injection, the animals were anesthetized for blood collection and then euthanized, and the concentration of total cholesterol in the blood serum was determined.

### 2.3. Data Analysis

The results of the study on the bioactive properties of mangiferin are presented using the average value of the sign value, X, and the error of the average value, SE. Since there were no more than 5 or 10 animals in each group when studying the anti-inflammatory and antidiabetic properties of the drug, respectively, when comparing the indicators of different groups, a nonparametric statistics method (Mann–Whitney test) was selected [74] as the most suitable for small samples. The presence of outliers was assessed according to the Grubbs’ test [75]. The normality of the sample distribution was checked using the Shapiro–Wilk test. The differences were considered statistically significant at *p* < 0.05.

## 3. Results and Discussion

One of the objectives of this study was to identify the correlation between the spatial structure of the mangiferin molecule and its biological activity.

### 3.1. Results of In Silico Analysis

#### 3.1.1. Prediction of the Spectrum of Targeted Mangiferin Activity in the Microcosm BioS System and Identification of Relevant Biotargets

**Figure 4:**
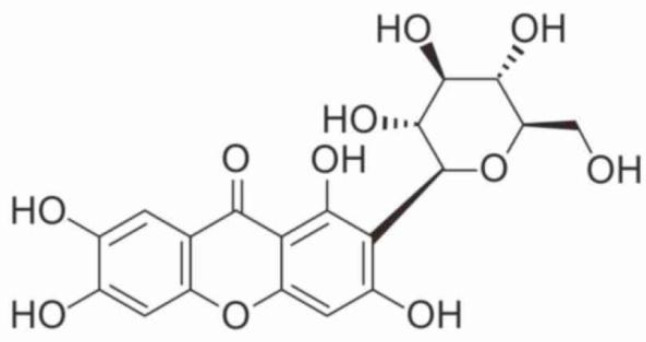
Structural formula of mangiferin.

When predicting the spectrum of targeted activity of the studied compound (Figure 4), 2697 types of targeted activity of *Homo sapiens* were processed in Microcosm BioS.

An analysis of the obtained data (Table 2) identified eight biotargets for which mangiferin was predicted to exhibit high activity (activity level index Ind ≥ +3.0) [76]. The targets are arranged in descending order of the overall activity level index. The final average theoretical activity values of mangiferin against the identified *Homo sapiens* biotargets were calculated. For comparative purposes, corresponding values for quercetin and rutin—flavonoids whose antidiabetic activity has been well-documented in multiple studies [77,78,79,80,81,82,83,84,85,86]—were calculated as well.

#### 3.1.2. Selection of 3D Models of Relevant Biotargets and Identification of Their Binding Sites

For the eight relevant biotargets, 605 experimental 3D X-ray diffraction models were found in the PDBe database [57]. After their analysis, in accordance with the criteria of validity, one of the most valid experimental 3D models was selected for each type of protein, which included one reference ligand. The general characteristics of the selected valid 3D models of *Homo sapiens* target proteins relevant to the spectrum of targeted activity of the studied compounds, and the key amino acids of specific binding sites of the eight relevant human target proteins, are shown in Table 3.

#### 3.1.3. Construction of Optimized 3D Models of the Studied Compound

**Figure 5:**
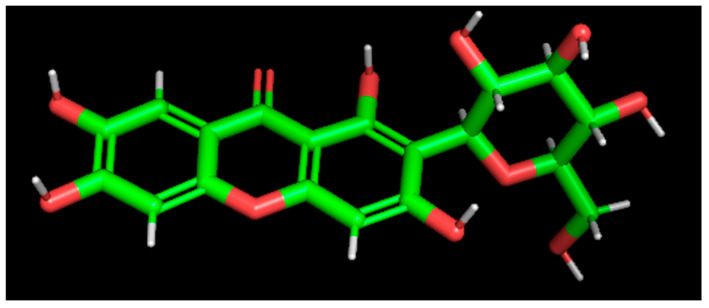
Mangiferin conformer with the lowest total energy.

Using molecular mechanics methods with the MarvinSketch 17.1.23 program, 10 conformers were constructed for the studied compound. They were optimized using the MOPAC2012 program, and the conformer with the lowest total energy was selected among them (Figure 5).

#### 3.1.4. Ensemble Docking of the Studied Compound into the Binding Sites of Relevant Biotargets and Determination of the Most Affine to the Studied Compound

As a result of processing an array of 2000 docking energy values for the compounds and each biotarget, the minimum docking energy, ΔE, was determined [53], the values of which are shown in Table 4.

Table 4 highlights the docking energy values for the biotargets most affine for the compound: *EGLN2, MAPK14, MAPK10, HCAR2, FABP4,* and *CALCRL.* At the same time, *MAPK10, HCAR2*, and *CALCRL*, which have high ΔE values, turned out to be the most “sensitive” to the studied compounds. According to the docking data, *FGF2* and *ATR* proteins are insufficiently affine for the studied compound and were not further considered in this study.

The compounds under consideration were characterized by a pronounced multitarget spectrum of activity (at least three of the most affine biotargets were found for them). According to the sum of the docking energies in the most affine biotargets, the multitarget potential decreased in the following order: rutin > mangiferin > quercetin, corresponding to the total values of the docking energy, −36.9, −30.2, and −27.6 kcal/mol. The highly affine biotargets *MAPK10*, *HCAR 2*, and *CALCRL* that were identified for the analyzed compounds play an important role in the regulation of apoptosis and the activation of inflammatory processes [87,88,89]. Thus, the spectrum of targeted activity of the studied compounds revealed in silico suggests that mangiferin should have a pronounced ability to reduce various kinds of inflammatory reactions.

#### 3.1.5. Analysis of the Molecular Mechanism of Binding of the Studied Compounds

**Figure 6:**
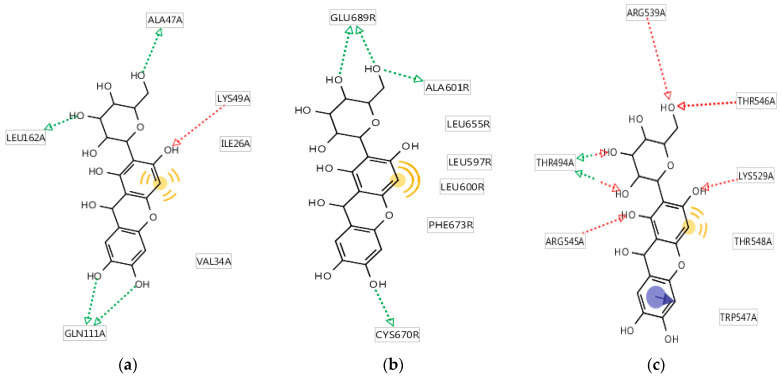
Characteristics of the binding of mangiferin to the affine biotarget sites: (**a**) *MAPK10*; (**b**) *HCAR2*; (**c**) *CALCRL*. Green arrows - hydrogen bonds of the donor type; red arrows - hydrogen bond of the acceptor type; yellow highlight - fragment participated in non-specific hydrophobic interactions; purple highlight – the benzene cycle forming stacking.

The results of the analysis of the molecular mechanism of binding of the studied compound to specific sites of the most affine biotargets are shown in Figure 6. It was found that five groups (Figure 6a) formed hydrogen bonds with ALA47, LYS49, GLN111, and LEU162 (four hydrogen bonds of the donor type, one hydrogen bond of the acceptor type); one fragment participated in non-specific hydrophobic interactions with ILE26, VAL34, and LEU162. Three groups (Figure 6b) formed donor-type hydrogen bonds with GLU689, ALA601, and CYS670; one fragment participated in non-specific hydrophobic interactions with LEU655, LEU597, LEU600, and PHE673. The benzene cycle formed stacking with TRP547; five groups formed hydrogen bonds with ARG539, THR546, LYS529, ARG545, and THR494 (six hydrogen bonds of the acceptor type, two hydrogen bonds of the donor type); one fragment participated in a non-specific hydrophobic interaction with LYS529 and THR548 (Figure 6c).

Quantitative indicators of the specific binding mechanisms of the studied compounds to the affine biotargets are summarized in a general table for a comparative analysis of the spectra of the molecular mechanisms of binding of the studied compound to the sites of the affine biotargets using nonparametric statistical methods (Table 5).

All three compounds considered together were different with high confidence (*p* = 0.0066, Kruskal–Wallis test). In a pairwise comparison (Mann–Whitney test), it was found that rutin differed significantly from quercetin in the spectra of the molecular binding mechanisms (*p* = 0.0486). In other cases, mangiferin did not differ statistically from either quercetin or rutin in its spectra of molecular binding mechanisms to the sites of the affine biotargets. Thus, it can be argued that the spectra of the molecular binding mechanisms of mangiferin, quercetin, and rutin are very similar.

#### 3.1.6. Consensus Prediction of Antiglycation Activity Using the IT Microcosm System

The prediction of the antiglycation activity of the studied compounds (Table 6) was carried out using conservative, normal, and risk forecasting strategies. The primary forecast estimates were then generalized within a two-level consensus, and the range of estimates was verified for consistency.

The results of the consensus prediction of the antiglycation activity of the studied compounds (Table 6) showed that mangiferin should exhibit a sufficiently pronounced activity in reducing the level of non-enzymatic protein glycation in diabetes mellitus less than quercetin but greater than rutin.

Thus, the in silico studies made it possible to determine the potential of mangiferin for anti-inflammatory activity and its ability to influence protein glycation, i.e., its antidiabetic potential. In the work of Aksenov and colleagues [90], the QSAR approach was used to predict the physicochemical properties and biologically active potential of mangiferin and its derivatives. The scientists predicted that neomangiferin has cardioprotective potential, mangiferin aglycone can be an inhibitor of the mitochondrial pathway for triggering apoptosis, and isomangiferin has antitumor activity. Noh and colleagues [11] presented in silico results in which mangiferin showed high binding affinity for inflammatory cytokines associated with macrophages and autophagy proteins, which does not contradict the results obtained in this study.

Next, the presence of the predicted anti-inflammatory and antidiabetic properties of mangiferin was tested in in vivo studies on rodents [91].

### 3.2. Results of In Vivo Experiment

#### 3.2.1. Anti-Inflammatory Activity In Vivo

Inflammation is the body’s response to the action of various damaging factors, which is a protective reaction of the body. Signs of an inflammatory reaction include redness, swelling, fever, soreness, and impaired function. However, inflammatory reactions can lead to profound morphological and functional disorders of organs and tissues [92,93].

The results obtained by modeling acute exudative inflammation are presented in Table 7.

Subplantar administration of λ-carrageenan initiated aseptic inflammation of the rat limb through activation of TLR4 receptors of immunocompetent cells. Group 1 was the negative control in this series of experiments, and Group 4 was the positive control. Compared to the controls, the condition of the animals after the administration of mangiferin was statistically significantly different; mangiferin at doses of both 50.0 mg/kg and 100.0 mg/ kg significantly increased edema. However, for Group 2, the value of *p* = 0.06 makes it practically a borderline case and warrants detailed study. The analysis of variance did not reveal a significant difference in the effect of mangiferin at a concentration of 50.0 mg/kg either 3 h or 4 h after modeling inflammation, but the administration of 100.0 mg/kg significantly increased edema after 3 h compared with the condition after 3 h. In scientific peer-reviewed sources [94], there is evidence of the presence of some pro-inflammatory activity in mangiferin. Compared to the positive control (diclofenac sodium, a nonsteroidal anti-inflammatory drug), mangiferin significantly increased edema by 31–32%.

The results obtained by modeling chronic proliferative inflammation are presented in Table 8.

The implanted cotton swabs caused granulomatous inflammation, and a week after implantation, they became covered with granulomatous tissue. Upon drying, the obtained granulomas lost a significant part of their mass, which was exudate. Group 5 was the negative control in this series of experiments, and Group 8 was the positive control. Compared to the negative control, the condition of the animals after the administration of mangiferin (50.0 mg/kg and 100.0 mg/kg) did not differ significantly (*p* > 0.05), but it differed significantly from the positive control group. Mangiferin at doses of 50.0 and 100.0 mg/kg had no effect on exudate weight. Compared to diclofenac sodium (positive control), mangiferin in Groups 6 and 7 significantly increased the exudation and proliferation of granulomatous tissue by 39 and 44%, respectively.

#### 3.2.2. Antidiabetic Activity In Vivo

The results of measuring the body weight of animals during the experiment to study hypoglycemic activity are shown in Table 9.

In the study on hypoglycemic activity in modeling diabetes mellitus, 16 rats died due to the toxic effect of a single intraperitoneal injection of an alloxan solution at a dose of 150.0 mg/kg [68]. The sample size was sufficient for statistical processing and did not contradict ethical principles [95]. The administration of mangiferin at all doses did not cause severe health problems or the death of animals. The dynamics of body weight in male rats in the diabetes mellitus model after the administration of mangiferin at doses of 50.0 and 100.0 mg/kg were comparable (*p* > 0.05) to those in the control groups (Group 9—group of intact animals; Group 10—negative control; Group 13—positive control). In rats with diabetes mellitus in Groups 11 and 12, body weight was comparable to that in Group 13, which received GLB at a dose of 5.0 mg/kg for 7 days (*p* > 0.05). A single intraperitoneal injection of alloxan at a dose of 150.0 mg/kg and an injection of mangiferin into the stomach at doses of 50.0 and 100.0 mg/kg did not affect the body weight dynamics of experimental animals.

In the study by Bidzhieva [96], no increase in body weight was observed in rats with alloxan-induced diabetes. On the contrary, the animals showed a decrease in body weight. Similar results—the absence of weight gain in rats with alloxan-induced diabetes—were observed in the studies by Cherkasova et al. [97], Volkhina et al. [98], Shaheen [99], and Amare et al. [100]. This research showed that body weight did not change in rats with alloxan-induced diabetes.

The results of the biochemical parameters of rat blood samples after the administration of mangiferin during the experiment to study hypoglycemic activity (glucose and cholesterol levels in the blood) are presented in Table 10.

Before the administration of alloxan (formation of a diabetes model), the concentration of glucose in the blood serum of intact animals (5.264 ± 0.374 mmol/l, n = 10) and the pool of animals for the formation of groups (5.254 ± 0.163 mmol/l, n = 40) corresponded to the norm. The values of these indicators did not differ (*p* > 0.05). Diabetes mellitus in rats developed 48 h after a single intraperitoneal injection of alloxan solution at a dose of 150.0 mg/kg (*p* < 0.05), as confirmed by increases in the concentrations of glucose and cholesterol in the blood serum of rats compared with intact animals. The effect persisted in experimental animals throughout the experiment (*p* < 0.05).

The administration of mangiferin at doses of 50.0 and 100.0 mg/kg into the stomach of experimental rats for 7 days did not lead to decreases in the concentrations of glucose and cholesterol in the blood serum (*p* > 0.05). GLB at a dose of 5.0 mg/kg reduced serum glucose and cholesterol concentrations, but not to the level of intact animals (*p* < 0.05). Under these experimental conditions, it was not possible to confirm the presence of hypoglycemic activity in mangiferin at doses of 50.0 and 100.0 mg/kg.

The delayed effect of mangiferin, which was isolated from *Hedysarum neglectum* with a degree of purification of at least 95%, is presumably due to its low bioavailability. The bioavailability of secondary metabolites remains an urgent area of research. Studying the bioavailability of plant polyphenols, including their release, absorption, distribution, metabolism, and excretion from the body, is crucial for understanding and optimizing their physiological functions. Phenolic compounds are released from the food matrix, absorbed, and then transported into the circulatory system through the epithelium of the gastrointestinal tract and eventually enter various tissues and organs, where they exert physiological effects. In order to maximize the beneficial properties of plant polyphenols, including mangiferin, it is necessary to study their digestibility and absorbability, as well as address problems related to bioavailability, an important indicator of nutrient availability influenced by various internal factors, including age, intestinal absorption capacity, health status, and genetics [101,102,103].

#### 3.2.3. Hypocholesterolemic Activity In Vivo

The results of blood biochemistry in mice during the assessment of the hypocholesterolemic activity of mangiferin are presented in Table 11.

With intraperitoneal administration of an aqueous solution of poloxamer P 407 (kolliphor) (a lipoprotein lipase inhibitor that disrupts lipoprotein clearance) at a dose of 400.0 mg/kg three times a week for 14 days, mice developed hypercholesterolemia accompanied by high blood cholesterol levels compared with intact animals (Group 14). The administration of mangiferin at doses of 50.0 and 100.0 mg/kg into the stomach of experimental mice for 14 days did not significantly change the concentration of cholesterol in the blood serum compared with the negative control group (intact animals).

In the course of in vivo studies on rodents, it was found that mangiferin at doses of 50.0 mg/kg and 100.0 mg/kg did not exhibit anti-inflammatory properties and hypoglycemic or hypocholesterolemic activity (Table 7, Table 8, Table 9, Table 10 and Table 11). These anti-inflammatory and antidiabetic activities have been predicted in silico and presented in the scientific literature. In a study by Noh and colleagues [11], it was shown that mangiferin (at a dose of 150.0 mg/kg) normalized body weight, organ weight, insulin sensitivity, glucose, and lipid metabolism in male C57BL/6 mice with induced obesity. Suman and colleagues [104] showed that Wistar rats with diabetes mellitus showed normalization of glucose, lipids, and insulin levels after 6 weeks of 40 mg/kg mangiferin administration. In addition, mangiferin had an anti-inflammatory effect [23].

As noted by Wang et al. [105] and Khan et al. [106], mangiferin demonstrates poor solubility (0.111 mg/mL) and extremely low oral bioavailability (1.2%). Like other flavonoids, particularly xanthones, mangiferin exhibits inherently low lipophilicity due to its chemical structure and extensive glycosidic bonding [107]. These structural characteristics also contribute to poor intestinal membrane permeability and limited absorption following oral administration. All the experimental and in vivo data obtained in the present study show that, despite the wide range of pharmacological activity, mangiferin has low solubility, transmembrane permeability, and bioavailability, which limit its clinical development and use.

Mangiferin, found in many plant species and isolated in large quantities from various parts of *Mangifera indica* (mango), has been well studied as a substance with powerful antioxidant, antidiabetic, antitumor, radioprotective, neuroprotective, anti-inflammatory, and other properties. The wide range of pharmacological properties of mangiferin, proven by many studies, provides a broad field for research and the development of medicines based on this research.

Mangiferin belongs to the C-glucosides, which contain C-glucoside bonds. The glycosyl substituent has antioxidant properties due to its structure and location relative to the aglycone fragment, and bioavailability is increased due to the glucose fragment. However, because of the glucose fragment, bioavailability is linked to hydrophilic properties, which do not ensure maximum transmembrane permeability with oral administration of mangiferin, as observed in the in vivo experiment on antidiabetic activity. This may explain the discrepancies between the experimental results of the in silico and in vivo models presented in this paper.

Mangiferin is mainly obtained from mango, along with many other active ingredients, but it has poor hydrophilicity and lipophilicity. Using RP-HPLC, the concentration of mangiferin was determined in rats that were orally administered with crude mangiferin and the mangiferin–phospholipid complex [108]. The results showed that the solubility of the mangiferin–phospholipid complex in water and n-octanol was improved 6.2-fold. For mangiferin isolated from *Hedysarum neglectum* hairy roots, the octanol–water partition coefficient was determined to be 0.43. According to Lipinski’s empirical rule [109], the distribution coefficient (log P) should not exceed 5. Based on the results obtained, mangiferin should exhibit the properties of biologically (pharmacologically) active compounds. The coefficient calculated by the degree of transition of a substance from the aqueous phase to n-octanol or from n-octanol to water makes it possible to predict biological activity.

Presumably, the reason for the in vivo results obtained in this study, which were inconsistent with the in silico results, is the need to reduce the dose of mangiferin in the current experimental conditions to less than 50 mg/kg (the results presented in Table 7 indicated a threshold probability value in the analysis of variance). Another reason could be the incompleteness of the group of considered markers of inflammation and diabetes, corresponding to the expression of genes such as *Caspase-1*, *Caspase-11*, *GSDMD*, etc., involved in various inflammatory reactions [110]. Nevertheless, the in vivo results, which do not correspond to the in silico predictions, contribute to the evidence base for the presence/absence of the anti-inflammatory, hypoglycemic, and hypocholesterolemic properties of mangiferin. The results of the present study provide information for further research and the development of technologies for medicines based on mangiferin, as well as open up new opportunities for the industrial production of mangiferin in vitro.

Research aimed at finding and using safe and active antidiabetic agents, such as bioavailable plant metabolites, is relevant and in demand due to the presence of side effects (edema, lactic acidosis, weight gain, hypoglycemia, etc.) associated with synthetic drugs [100].

## 4. Conclusions

In an in silico study, it was found that mangiferin is capable of exhibiting a multi-target effect. According to the revealed spectrum of targeted activity, it is suggested that mangiferin should have a pronounced ability to inhibit various kinds of inflammatory reactions. However, in in vivo studies of mangiferin in acute exudative inflammation caused by λ-carrageenan in rats, mangiferin at doses of 50.0 and 100.0 mg/kg with a single injection into the stomach under these experimental conditions increased the amount of edema, i.e., had a pro-inflammatory effect on acute inflammation. In chronic proliferative inflammation caused by the implantation of cotton swabs under the skin of rats, the same doses of mangiferin did not affect the proliferative response. In an in silico study using the IT Microcosm system, it was found that mangiferin should be quite active in reducing the level of non-enzymatic protein glycation in diabetes mellitus. In vivo studies in rodents showed that in a model with alloxan-induced diabetes mellitus in rats with 7-fold administration and in a model of poloxamer-induced hypercholesterolemia in mice with 14-fold administration, mangiferin did not have hypoglycemic or hypocholesterolemic activity at doses of 50.0 and 100.0 mg/kg. In the framework of further research, attention should be paid to the presence of the anti-inflammatory effects of mangiferin at concentrations not exceeding 50.0 mg/kg, and to the presence of antidiabetic potential in mangiferin at alternative doses (from 5 to 50 and from 150 to 200 mg/kg). Studies will be carried out aimed at increasing the bioavailability of mangiferin, which in the long term will facilitate obtaining in vivo results corresponding to the in silico results. For example, derivatization of mangiferin by complexing it with cyclic oligosaccharides, obtaining mangiferin derivatives using esterification and aryl-alkylation reactions, obtaining metal complexes, and using exosomal and liposomal nanocarriers [111,112].

## Figures and Tables

**Table 1:** Design of the study on the biological activity of mangiferin in vivo models.

Group Number	Number of Animals	Model	Injected Substance			
			Water, mL	Mangiferin, mg/kg	Diclofenac Sodium, mg/kg	GLB, mg/kg
1	5	A	1.0	-	-	-
2	5	A	-	50.0	-	-
3	5	A	-	100.0	-	-
4	5	A	-	-	10.0	-
5	5	B	1.0	-	-	-
6	5	B	-	50.0	-	-
7	5	B	-	100.0	-	-
8	5	B	-	-	10.0	-
9	10	-	-	-	-	-
10	10	C	1.0	-	-	-
11	10	C	-	50.0	-	-
12	10	C	-	100.0	-	-
13	10	C	-	-	-	5.0
14	5	-	-	-	-	-
15	8	D	1.0	-	-	-
16	8	D	-	50.0	-	-
17	8	D	-	100.0	-	-

A—acute inflammation “carrageenan edema”; B—acute inflammation “cotton granuloma”; C—diabetes mellitus; D—hypercholesterolemia; GLB—glibenclamide. Groups 1, 5, 10, 15 —negative control; Groups 4, 8, 13 —positive control; Groups 9, 14 —intact animals (healthy animals that were kept and fed under general conditions, without treatment).Adaptation and quarantine of animals. Before the experiment, the animals underwent adaptation for 3 days. Quarantine of animals was not provided.

**Table 2:** The range of levels of targeted mangiferin activities in relation to the relevant *Homo sapiens* biotargets.

Target	Gene ^1^	Medium group		Mangiferin		Quercetin		Rutin	
		T	Ind	T	Ind	T	Ind	T	Ind
Hypoxia-inducible factor prolyl hydroxylase 1	*EGLN2*	0.26	3.80	0.24	4.8 **	0.27	3.1	0.27	3.5
Mitogen-activated protein kinase 14	*MAPK14*	0.55	3.57	0.39	2.3 **	0.68	3.7	0.59	4.7
Basic fibroblast growth factor	*FGF2*	0.18	3.43	0.17	2.4 **	0.15	3.6	0.23	4.3
Mitogen-activated protein kinase 10	*MAPK10*	0.52	3.37	0.35	1.5 **	0.64	4.3	0.57	4.3
Serine/threonine-protein kinase atr	*ATR*	0.28	3.37	0.30	3.7 *	0.29	2.8	0.26	3.6
Hydroxycarboxylic acid receptor 2	*HCAR2*	0.28	3.37	0.30	3.7 *	0.29	2.8	0.26	3.6
Fatty acid binding protein adipocyte	*FABP4*	0.24	3.30	0.26	3.6	0.24	3.0	0.21	3.3
Calcitonin gene-related peptide type 1 receptor	*CALCRL*	0.32	2.70	0.35	−0.7 **	0.29	4.4	0.32	4.4

^1^ *EGLN2* participates in the regulation of apoptosis in cardiac and skeletal muscles; *MAPK10* participates in the regulation of neuronal apoptosis; *ATR* is responsible for the activation of kinases; *HCAR2* mediates apoptosis induced by nicotinic acid; *FGF2* is a fibroblast growth factor; *CALCRL* participates in the regulation of apoptosis of endothelial cells in the microcirculatory system; *MAPK14* participates in the induction of inflammatory cytokines; *FABP4* delivers long-chain fatty acids and retinoic acid to related receptors in the nucleus that activate various expression mechanisms. T is the average QL-modified Tanimoto similarity coefficient; Ind is the average activity level index. The * or ** symbol indicates values that differ significantly from those of quercitin or quercetin and rutin, respectively (*p* < 0.05, Mann–Whitney test).

**Table 3:** Valid experimental 3D models of human target proteins relevant to the spectrum of targeted activity of the studied natural compounds and the key amino acids of specific binding sites of the relevant biotargets.

Biotarget ^1^	Name ^2^	PDBe ^3^	Code ^4^	Key Amino Acids of the Site
*EGLN2*	Prolyl hydroxylase EGLN2	1	5v1b	TYR287, TYR294, HIS297, ILE311, TYR313, ASN315, HIS358, VAL360, ARG367, ALA369
*MAPK14*	Mitogen-activated protein kinase 14	245	2bal	ALA51, LYS53, LEU104, THR106, HIS107, LEU108, MET109, GLY110, ALA111
*FGF2*	Fibroblast growth factor 2	22	2fgf	LYS26, ASN27, ASN101, LYS119, ARG120, LYS125, GLN134, LYS135, ALA136
*MAPK10*	Mitogen-activated protein kinase 10	60	2o0u	ILE70, VAL78, ALA91, LYS93, MET146, GLU147, LEU148, MET149, ASP150, ALA151, ASN152, GLN155, VAL196, LEU206
*ATR*	Serine/threonine-protein kinase ATR	1	5yz0	LYS2308, ILE2377, TRP2379, VAL2380, ASN2381, THR2383, PRO2388, ASN2480, ILE2481, VAL2493, ASP2494
*HCAR2*	Hydroxycarboxylic acid receptor 2	13	7xk2	LEU83, LEU104, LEU107, ALA108, ARG111, GLN112, LEU158, LEU162, SER179, PHE180, HIS189, MET192, PHE193, PHE277, LEU280, TYR284
*FABP4*	Fatty acid-binding protein, adipocyte	240	2hnx	PHE16, TYR19, MET20, VAL25, ALA33, ALA36, PRO38, SER53, PHE57, ALA75, ILE104, VAL115 ARG108, TYR128, ARG130
*CALCRL*	Calcitonin gene-related peptide type 1 receptor	23	8ax7	ASP1071, TRP1074, TRP1084, ARG2038, ASP2070, GLY2071, TRP2072, TRP2121, THR2122, TYR2124
Total		605		

^1^ The designation of the gene and protein recommended by UniProt [56]. ^2^ The standard name of the protein recommended by UniProt [56]. ^3^ The number of experimental 3D models found in PDBe [57]. ^4^ The PDB code of the selected valid 3D model.

**Table 4:** Docking energy of the studied natural compounds into specific binding sites of the relevant biotargets.

Compound	Docking Energy in Biotarget, kcal/mol							
	*EGLN2*	*MAPK14*	*FGF2*	*MAPK10*	*ATR*	*HCAR2*	*FABP4*	*CALCRL*
Mangiferin	−8.8	−8.9	−5.6	−10.0	−7.3	−10.1	−7.4	−10.1
Quercetin	−9.2	−8.8	−5.5	−9.3	−7.0	−9.1	−8.7	−8.4
Rutin	−8.8	−9.3	−5.6	−9.1	−8.1	−6.3	−9.2	−9.3

**Table 5:** Features of the molecular mechanisms of binding of the studied compounds to the sites of the affine biotargets.

Biotarget	Type of Binding	Number of Bonds		
		Mangiferin	Quercetin	Rutin
*MAPK10*	HD	4	0	7
	HA	1	1	6
	NS	1	1	2
	St	0	0	0
*HCAR2*	HD	3	2	—
	HA	0	3	—
	NS	1	1	—
	St	0	0	—
*CALCRL*	HD	6	—	5
	HA	2	—	3
	NS	1	—	2
	St	1	—	0

HD—hydrogen bonding of the donor type; HA—hydrogen bonding of the acceptor type; NS—non-specific hydrophobic interactions; St—stacking.

**Table 6:** Consensus prediction of the second level of antiglycation activity of the studied compounds according to a set of strategies.

Compound	Assessment of the Activity Level by Strategy			
	Conservative	Normal	Risk	General
Mangiferin	moderate	moderate	moderate	moderate
Quercetin	moderate	moderate	high	high? ^1^
Rutin	moderate	moderate	low	moderate?

^1^ The symbol “?” the simulation results indicate the ambiguity of the conclusion, which requires further investigation.

**Table 7:** Average data on the severity of paw edema in animals (X ± SE).

Group Number	Edema Size, μL	
	After 3 h	After 4 h
1	480 ± 50	480 ± 20
2	600 ± 60 *	600 ± 30 *
3	610 ± 40 *	740 ± 90 *
4	340 ± 30	330 ± 40

Group 1—negative control; Group 2—received mangiferin (50.0 mg/kg); Group 3—received mangiferin (100.0 mg/kg); Group 4—positive control. The values in the columns marked with the * symbol differed significantly from those of the control groups (*p* < 0.05, Mann–Whitney test).

**Table 8:** Average data on the severity of chronic proliferative inflammation (X ± SE).

Group Number	Mass of Exudate, mg	Mass of Granulation Tissue, mg
5	138.3 ± 10.9	40.6 ± 3.3
6	149.2 ± 13.8 *	43.7 ± 3.4 *
7	144.9 ± 9.7 *	38.1 ± 2.7 *
8	84.1 ± 5.3	22.9 ± 1.3

Group 5—negative control; Group 6—received mangiferin (50.0 mg/kg); Group 7—received mangiferin (100.0 mg/kg); Group 8—positive control. The values in the columns marked with the * symbol differed significantly from those of the positive control group (*p* < 0.05, Mann–Whitney test).

**Table 9:** Average body weight data of rats after the administration of mangiferin and GLB (X ± SE).

Time Point	Animal Group Number				
	9	10	11	12	13
0	242 ± 4	243 ± 1	243 ± 1	243 ± 1	243 ± 1
	(n = 10)	(n = 10)	(n = 10)	(n = 10)	(n = 10)
1	243 ± 4	246 ± 4	243 ± 4	243 ± 3	247 ± 3
	(n = 10)	(n = 10)	(n = 10)	(n = 10)	(n = 10)
2	247 ± 4	248 ± 2	246 ± 4	246 ± 4	248 ± 5
	(n = 10)	(n = 9)	(n = 9)	(n = 8)	(n = 9)
3	249 ± 3	251 ± 4	248 ± 4	249 ± 3	248 ± 4
	(n = 10)	(n = 7)	(n = 7)	(n = 6)	(n = 7)
4	258 ± 3	260 ± 4	258 ± 5	257 ± 2	257 ± 4
	(n = 10)	(n = 6)	(n = 6)	(n = 6)	(n = 6)

Group 9—intact animals (healthy animals that were kept and fed under general conditions, without treatment); Group 10—negative control; Group 11—received mangiferin (50.0 mg/kg); Group 12—received mangiferin (100.0 mg/kg); Group 13—positive control. 0—before administration; 1—48 h after alloxan administration; 2—1 week after alloxan administration; 3—2 weeks after alloxan administration; 4—3 weeks after alloxan administration. All values in the rows did not differ significantly from those of the control groups (Groups 9 and 13) (*p* > 0.05, Mann–Whitney test).

**Table 10:** The average values of glucose and cholesterol levels in the blood of rats during the experiment (X ± SE).

Time Point	Animal Group Number				
	9	10	11	12	13
	Glucose, mmol/L				
0	5.26 ± 0.37	5.25 ± 0.16	5.25 ± 0.16	5.25 ± 0.16	5.25 ± 0.16
	(n = 10)	(n = 10)	(n = 10)	(n = 10)	(n = 10)
1	4.88 ± 0.30	22.62 ± 0.79	22.57 ± 0.52	21.67 ± 1.21	21.48 ± 0.62
	(n = 10)	(n = 10)	(n = 10)	(n = 10)	(n = 10)
2	4.34 ± 0.23	24.46 ± 0.70	25.36 ±0.79	25.91 ± 0.91	25.13 ± 0.58
	(n = 10)	(n = 9)	(n = 9)	(n = 8)	(n = 9)
3	4.53 ± 0.29	23.37 ± 0.72	23.70 ± 0.68	23.26 ± 0.57	22.86 ±0.32
	(n = 10)	(n = 7)	(n = 7)	(n = 6)	(n = 7)
4	4.37 ± 0.38	20.09 ± 0.40	20.44 ± 0.28 *	21.12 ± 0.43*	13.02 ± 0.58
	(n = 10)	(n = 6)	(n = 6)	(n = 6)	(n = 6)
	Cholesterol, mmol/L				
0	1.63 ± 0.06	1.62 ± 0.06	1.63 ± 0.06	1.63 ± 0.06	1.62 ± 0.06
	(n = 10)	(n = 10)	(n = 10)	(n = 10)	(n = 10)
2	1.61 ±0.06	2.59 ± 0.09	2.62 ± 0.13 *	2.61 ± 0.07 *	2.58 ±0.04
	(n = 10)	(n = 9)	(n = 9)	(n = 8)	(n = 9)
3	1.54 ± 0.09	2.58 ± 0.07	2.46 ± 0.07 *	2.53 ± 0.11 *	2.55 ± 0.07
	(n = 10)	(n = 7)	(n = 7)	(n = 6)	(n = 7)
4	1.58 ± 0.07	2.44 ±0.07	2.46 ± 0.10 *	2.43 ± 0.14 *	1.93 ± 0.04
	(n = 10)	(n = 6)	(n = 6)	(n = 6)	(n = 6)

Group 9—intact animals (healthy animals that were kept and fed under general conditions, without treatment); Group 10—negative control; Group 11—received mangiferin (50.0 mg/kg); Group 12—received mangiferin (100.0 mg/kg); Group 13—positive control. 0—before administration; 1—48 h after alloxan administration; 2—1 week after alloxan administration; 3—2 weeks after alloxan administration; 4—3 weeks after alloxan administration. The values in the rows marked with the * symbol differed significantly from those of the group of intact animals, Group 9 (*p* < 0.05, Mann–Whitney criterion).

**Table 11:** Average parameters of blood biochemistry in mice after oral administration of mangiferin at doses of 50 and 100 mg/kg.

Indicator, Units	Groups of Animals			
	14	15	16	17
Cholesterol, mmol/l	1.921 ± 0.199	9.923 ± 1.274	10.706 ± 1.023 *	10.626 ± 1.489 *

Group 14—intact animals (healthy animals that were kept and fed under general conditions, without treatment); Group 15—negative control; Group 16—received mangiferin (50.0 mg/kg); Group 17—received mangiferin (100.0 mg/kg). The values in the rows marked with the * symbol differed significantly from those of the group of intact animals, Group 14 (*p* < 0.05, Mann–Whitney criterion).

## Data Availability

Further inquiries can be directed to the corresponding author.
